# Comparison of Quadratus Lumborum Block and Rectus Sheath Block for Postoperative Analgesia in Single-Port Laparoscopic Adnexal Surgery: A Randomized Controlled Trial

**DOI:** 10.3390/medicina61061084

**Published:** 2025-06-13

**Authors:** Dongju Kim, Seunguk Bang, Jihyun Chung, Youngin Lee, Hyun-Jung Shin, Yoonji Park

**Affiliations:** 1 Department of Surgery, Daejeon St. Mary’s Hospital, College of Medicine, The Catholic University of Korea, Seoul 03083, Republic of Korea; reallydong@gmail.com; 2 Department of Anesthesiology and Pain Medicine, Daejeon St. Mary’s Hospital, College of Medicine, The Catholic University of Korea, Seoul 03083, Republic of Korea; anesth126@catholic.ac.kr (J.C.); technobear@naver.com (Y.L.); pyj5648@naver.com (Y.P.); 3 Department of Anesthesiology and Pain Medicine, Seoul National University Bundang Hospital, Seongnam 13620, Republic of Korea; 4 Department of Anesthesiology and Pain Medicine, Seoul National University College of Medicine, Seoul 03080, Republic of Korea

**Keywords:** hysterectomy, laparoscopic, nerve block, postoperative pain, quadratus lumborum block, single-port

## Abstract

*Background and Objectives*: Regional anesthesia is a key component of multimodal analgesia following minimally invasive gynecologic surgery. However, single-port laparoscopic adnexal surgery differs anatomically and physiologically from multiport or open approaches, particularly in terms of incision site, tissue handling, and pain characteristics. Despite its increasing use, evidence supporting procedure-specific regional analgesic protocols for this approach remains limited. This study aimed to compare the analgesic efficacy of quadratus lumborum block (QLB) and rectus sheath block (RSB) in this surgical context. *Materials and Methods*: In this randomized controlled trial, 68 patients undergoing single-port laparoscopic adnexal surgery were randomly assigned to receive either QLB or RSB at the end of surgery. Four patients were excluded due to missing patient-controlled analgesia (PCA) data, resulting in 64 patients analyzed (QLB group: *n* = 32; RSB group: *n* = 32). The primary outcome was cumulative opioid consumption over the first 24 postoperative hours. Secondary outcomes included interval-based opioid consumption, time to first PCA bolus, postoperative pain scores, and incidence of postoperative nausea and vomiting (PONV). *Results*: The RSB group demonstrated significantly lower cumulative opioid consumption at 24 h postoperatively (132.9 [61.3, 338.4] µg vs. 453.0 [253.1, 811.0] µg, *p* < 0.001). This trend persisted across most postoperative time points up to 48 h. Interval-based opioid consumption was also lower in the RSB group during 0–24 h and 32–48 h intervals (each comparison *p* < 0.05). The time to first PCA bolus was significantly longer in the RSB group (56.5 [41.0, 340.3] minutes vs. 40.5 [33.3, 68.8] minutes; *p* = 0.014), and Kaplan–Meier analysis confirmed a delayed first bolus request in the RSB group (log-rank *p* = 0.007). Pain scores and postoperative nausea and vomiting incidence were comparable between groups. *Conclusions*: Compared with QLB, RSB provided similar pain relief with significantly lower opioid consumption following single-port laparoscopic adnexal surgery. These findings highlight the potential advantages of RSB in enhancing analgesic efficiency and support the development of procedure-specific regional analgesia protocols tailored to this surgical approach.

## 1. Introduction

Minimally invasive surgery has become the standard approach for many gynecologic procedures, gradually evolving from open laparotomy to multiport laparoscopy, and more recently to single-port laparoscopy. As surgical techniques have become less invasive, the nature and intensity of postoperative pain have also changed. Applying traditional analgesic strategies developed for open surgeries to these minimally invasive procedures may result in overtreatment, akin to using a flamethrower to catch a flea. Thus, there is an increasing need for procedure-specific pain management strategies that are optimized to the surgical approach and its associated pain characteristics.

Multimodal analgesia is now widely regarded as a fundamental principle of postoperative pain control [1]. Among its components, regional anesthesia techniques play a crucial role by significantly reducing the somatic and sometimes visceral components of postoperative pain while minimizing opioid requirements [2]. In the context of obstetric and gynecologic surgery, including multiport laparoscopic procedures, regional techniques such as wound infiltration, the transversus abdominis plane (TAP) block, and the quadratus lumborum block (QLB) have been shown to improve postoperative analgesia and reduce opioid consumption [3,4,5]. However, evidence remains limited for single-port laparoscopic surgery, which differs anatomically and physiologically from multiport approaches in terms of port location, incision size, and resulting pain pattern. Although single-port access laparoscopy is classified as a minimally invasive technique, the single umbilical incision often requires expansion to accommodate multiple instruments through a single site. Typically, multiports are inserted through the umbilicus, and to ensure adequate maneuvering space, the incision is often widened beyond the size of standard 5 mm or 10 mm trocars—commonly to approximately 1.5 to 2 cm. Consequently, patients frequently report significant umbilical pain during the early postoperative period—often more severe than that experienced after conventional multiport laparoscopy—and tend to require higher doses of postoperative analgesics to manage this pain [6,7,8]. This distinctive pain profile suggests that procedure-specific strategies, such as abdominal wall blocks, may be particularly beneficial in single-port laparoscopic procedures and warrants focused investigation.

The postoperative pain pattern following adnexal surgery involves a combination of somatic and visceral pain. Somatic pain primarily arises from skin incisions at the port site, stretching or disruption of the muscle fascia, and irritation of the parietal peritoneum [9]. These structures are innervated by the lower thoracic spinal nerves, particularly T9–T11, which correspond to the periumbilical region. In contrast, visceral pain originates from the manipulation of pelvic organs such as the ovaries and fallopian tubes, which are innervated by autonomic pathways. Sympathetic fibers arise from the T10–L2 spinal segments and travel via the ovarian plexus and lumbar splanchnic nerves, whereas parasympathetic fibers arise from S2–S4 and travel via the pelvic splanchnic nerves [10,11]. While visceral pain tends to be dull and poorly localized, somatic pain is typically sharp and well-defined. In single-port laparoscopic adnexal surgery, somatic pain—particularly that arising from the umbilical incision—is generally regarded as the predominant component during the early postoperative phase.

The rectus sheath block (RSB) targets the anterior cutaneous branches of the intercostal nerves, typically at the T9–T11 level, providing effective somatic analgesia to the anterior abdominal wall. By blocking the nerve supply to the periumbilical skin and fascia, RSB is known to be highly effective for managing pain following umbilical hernia repair [12,13]. For this reason, RSB has also shown benefits in various single-port laparoscopic procedures by reducing postoperative somatic pain at the incision site and decreasing analgesic requirements [14]. However, due to its somatic-only mechanism, RSB does not affect autonomic fibers and therefore has no impact on visceral pain.

In contrast, the quadratus lumborum block (QLB), first described by Blanco et al. in 2016, is an extended version of the TAP block that provides both somatic and potentially visceral analgesia. QLB blocks the ventral rami of the thoracolumbar spinal nerves (T8–L1), thereby covering the somatic pain component. Additionally, local anesthetics may spread to the thoracic paravertebral space (TPVS), where they can modulate sympathetic fibers along the sympathetic chain. Through this mechanism, QLB may exert an analgesic effect on certain aspects of visceral pain [15]. Thus, QLB theoretically offers a broader analgesic range than RSB—covering not only the same somatic territories but also some visceral pathways via sympathetic blockade.

To our knowledge, no previous studies have directly compared the analgesic efficacy of QLB and RSB in the setting of single-port laparoscopic adnexal surgery. Accordingly, we conducted a randomized controlled trial to compare the two techniques. We hypothesized that because QLB can control somatic pain similarly to RSB while additionally alleviating visceral pain, it would provide greater opioid-sparing effects. The primary outcome was cumulative opioid consumption during the first 24 h postoperatively.

## 2. Materials and Methods

### 2.1. Study Design and Participants

This randomized, controlled, observer-blinded trial was conducted at Daejeon St. Mary’s Hospital, College of Medicine, The Catholic University of Korea, Republic of Korea. This study adhered to the ethical principles outlined in the Declaration of Helsinki. Ethical approval was obtained from the Institutional Review Board of The Catholic University of Korea (IRB No DC18EESI0018 and date of approval 12 April 2018). This study was prospectively registered with the Clinical Research Information Service (CRIS; KCT0002812 and date of approval 20 April 2018), a primary registry of the WHO International Clinical Trials Registry Platform. Written informed consent was obtained from all participants prior to study enrollment. A total of 68 female patients aged 18 to 65 years, scheduled for elective single-port laparoscopic gynecologic surgery for benign ovarian disease, were enrolled. Eligible patients were classified as ASA physical status I to III. Exclusion criteria included: malignancy, coagulopathy, infection at the injection site, allergy to local anesthetics, severe cardiopulmonary disease, BMI > 35, pre-existing neuropathy, chronic opioid use, inability to understand the patient-controlled analgesia (PCA) device, or refusal/preference for a specific nerve block technique.

### 2.2. Randomization

Patients were randomly assigned to receive either QLB or RSB in a 1:1 ratio using a computer-generated randomization sequence. Group allocation was concealed using opaque, sealed envelopes. The anesthesiologist performing the block was not involved in outcome assessment. Patients and the outcome assessor were blinded to group allocation.

### 2.3. Intraoperative Management

All patients underwent general anesthesia following a standardized protocol. Anesthesia induction was performed with intravenous propofol (2 mg/kg) and remifentanil, followed by neuromuscular blockade using rocuronium (0.6 mg/kg). After the loss of consciousness and confirmation of adequate muscle relaxation, endotracheal intubation was performed. Anesthesia was maintained with desflurane in a mixture of oxygen and air, along with continuous remifentanil infusion using the target-controlled infusion (TCI) mode (effect-site concentration 2–4 ng/mL), titrated to maintain hemodynamic stability. Neuromuscular blockade was reversed at the end of the surgery using pyridostigmine and glycopyrrolate, once a train-of-four ratio > 95% was confirmed.

As part of a perioperative multimodal analgesia protocol and prophylaxis against postoperative nausea and vomiting, all patients received dexamethasone (5 mg) after induction, ketorolac (30 mg), propacetamol (2 g), and ramosetron (0.3 mg), all administered approximately 30 min before the end of surgery.

Surgical procedures were performed via a single-port laparoscopic approach through a 1.5–2.0 cm transumbilical incision, using a multichannel port system. Pneumoperitoneum was established with carbon dioxide insufflation and maintained at a pressure of 10–12 mmHg. All operations were performed by the same surgical team specializing in gynecologic laparoscopy.

### 2.4. Block Technique

All regional blocks were performed under ultrasound guidance (WS80A, Samsung Medison, Seoul, Republic of Korea) by experienced anesthesiologists after the end of the surgery. Each block was performed under sterile conditions following skin preparation and sterile draping.

#### 2.4.1. QLB

**Figure 1:**
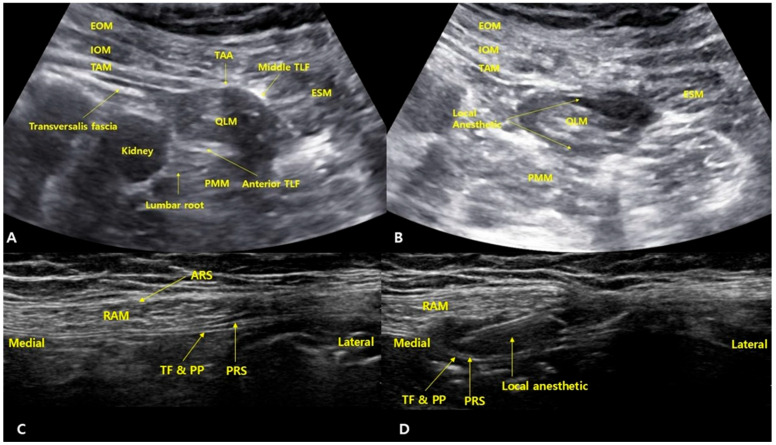
Ultrasound images illustrating the quadratus lumborum block (QLB) and rectus sheath block (RSB). (**A**) Before the injection of local anesthetic for QLB. (**B**) After injection, showing the spread of local anesthetic around the quadratus lumborum muscle, with visible accumulation between the middle and anterior layers of the thoracolumbar fascia. (**C**) Before the injection of local anesthetic for RSB. (**D**) After injection, the local anesthetic spreads between the posterior aspect of the rectus abdominis muscle and the posterior rectus sheath. ARS: anterior rectus sheath, EOM: external oblique muscle, ESM: erector spinae muscle, IOM: internal oblique muscle, PMM: psoas major muscle, PRS: posterior rectus sheath, QLM: quadratus lumborum muscle, RAM: rectus abdominis muscle, TAA: transversus abdominis aponeurosis, TAM: transversus abdominis muscle, TF and PP: transversalis fascia and parietal peritoneum, TLF: thoracolumbar fascia.

After the completion of surgery, patients were placed in a slightly tilted supine position with a folded pillow under the buttocks to create sufficient space for the ultrasound probe. A convex probe was placed along the midaxillary line and scanned posteromedially to identify the three abdominal wall muscles and the aponeurosis of the transversus abdominis. The thoracolumbar fascia (TLF) was identified, including the anterior layer surrounding the psoas major, the middle layer surrounding the quadratus lumborum, and the posterior layer around the erector spinae muscles. A 22-gauge Tuohy needle was inserted from the anterolateral aspect toward the posteromedial direction, targeting the middle layer of the thoracolumbar fascia adjacent to the lumbar interfascial triangle area. After the needle tip contacted the middle TLF, a tactile “pop” was felt as it passed through. A small volume of saline (1–2 mL) was injected to confirm that the needle tip was correctly positioned in the fascial plane between the middle TLF and the QLM. Then, 20 mL of 0.375% ropivacaine with epinephrine (5 µg/mL) was administered (Figure 1). The probe was then rotated 90 degrees to confirm that the local anesthetic had spread between the middle and anterior layers of the TLF, surrounding the quadratus lumborum muscle (QLM). The same procedure was performed on the contralateral side.

#### 2.4.2. RSB

After the completion of surgery, with the patient in the supine position, a linear ultrasound probe was placed at the level of the umbilicus. After identifying the linea alba, the probe was moved laterally to visualize the anterior rectus sheath, rectus abdominis muscle, posterior rectus sheath, transversalis fascia, and parietal peritoneum. Further lateral scanning was performed to confirm the three abdominal wall muscles and the linea semilunaris. As the anterior cutaneous branches of the intercostal nerves may branch off laterally before reaching the midline, injection too medially may lead to incomplete blockade. Therefore, the injection site was selected at the lateral one-fourth of the rectus abdominis muscle, just medial to the linea semilunaris, where the posterior rectus sheath begins to envelop the muscle. A 22-gauge needle was inserted in-plane into the space between the posterior rectus sheath and the rectus abdominis muscle. Correct needle placement was confirmed with 1–2 mL of saline, followed by an injection of 20 mL of 0.375% ropivacaine with epinephrine (5 µg/mL). The probe was then rotated 90 degrees to confirm the spread of the local anesthetic between the posterior rectus sheath and rectus abdominis muscle. The same procedure was repeated on the contralateral side.

### 2.5. Postoperative Analgesia

All patients received a fentanyl-based PCA (Accumate 1200, Woo Young Medical, Daegu, Republic of Korea) in bolus-only mode (0.5 µg/kg per bolus, 7-min lockout, maximum 4 µg/kg every 4 h). Oral celecoxib and acetaminophen were administered as scheduled postoperative analgesics. Tramadol 25 mg IV was administered as rescue analgesia if the visual analogue scale (VAS) > 4. For the analysis of cumulative opioid consumption, tramadol was converted to fentanyl equivalents using a 1:1000 conversion ratio (50 mg tramadol = 50 µg fentanyl), as commonly reported in the literature and supported by evidence from multiple randomized controlled trials [16,17].

### 2.6. Outcome Measures

The primary outcome was cumulative opioid consumption over the first 24 h postoperatively. Secondary outcomes included cumulative and interval opioid consumption at various time points (2, 4, 8, 12, 18, 24, 32, and 48 h), time to first PCA bolus, pain scores (VAS), and the incidence of postoperative nausea and vomiting (PONV). We used the Apfel simplified risk score to assess the risk of PONV. The Apfel score includes four independent predictors: The time to first PCA bolus was defined as the time when the patient first pressed the PCA button. In this study, sensory blockade was evaluated in the post-anesthesia care unit (PACU) approximately one hour after the administration of the nerve blocks. The sensory testing involved pinprick stimulation across the T4–L3 dermatomes. A four-point grading scale was used to assess the sensory response. A score of 0 indicated normal sensation, meaning that cold, pain, and touch perception were identical to those in non-affected areas such as the neck or upper limbs. A score of 1 represented hypoalgesia, in which all sensations were present but reduced compared to unaffected body parts. A score of 2 indicated complete loss of cold and pain sensation while touch perception remained intact. A score of 3 signifies complete loss of cold, pain, and touch sensation. The nerve block was considered technically successful if at least one dermatome between T4 and L3 demonstrated a sensory grade of 1 or higher. If none of the assessed dermatomes showed a score above 0, the block was classified as a technical failure and excluded from the final analysis. This evaluation procedure was applied to both the QLB and the RSB group.

Immediately after the block procedure, real-time US imaging was used to verify correct injectate spread. For QLB, the transducer was rotated 90 degrees to obtain a sagittal view, and the spread of local anesthetic between the QLM and the middle layer of TLF, as well as between the QLM and the anterior TLF, was confirmed. If these anatomical targets were appropriately visualized with local anesthetic spread, the block was not considered a technical error. Similarly, for RSB, the local anesthetic was confirmed to spread between the posterior rectus sheath and the rectus abdominis muscle under ultrasound guidance. When this pattern of spread was observed, the block was likewise regarded as technically successful.

### 2.7. Sample Size

Sample size estimation was based on a previous study, which reported a 24-h postoperative opioid consumption of 78 mg (interquartile range: 61–90) in patients undergoing laparoscopic gynecologic surgery [18]. Using this reference, we assumed a mean of 78 mg and an estimated standard deviation (SD) of 21.64 mg. A 20% reduction in opioid consumption (15.6 mg) was considered clinically meaningful. With a two-sided α of 0.05 and power (1–β) of 80%, the required sample size was calculated to be 30.2 per group. Allowing for a 10% dropout rate, 34 patients were enrolled in each group, resulting in a total sample size of 68.

### 2.8. Statistical Analysis

Statistical analyses were conducted using Statistical Package for the Social Sciences (SPSS) version 20. Continuous variables were analyzed using Student’s *t*-test or Mann–Whitney U test [10]. Categorical variables were assessed with chi-square or Fisher’s exact test. Time to first PCA bolus was analyzed using Kaplan–Meier curves and log-rank test. A *p*-value < 0.05 was considered statistically significant.

## 3. Results

**Figure 2:**
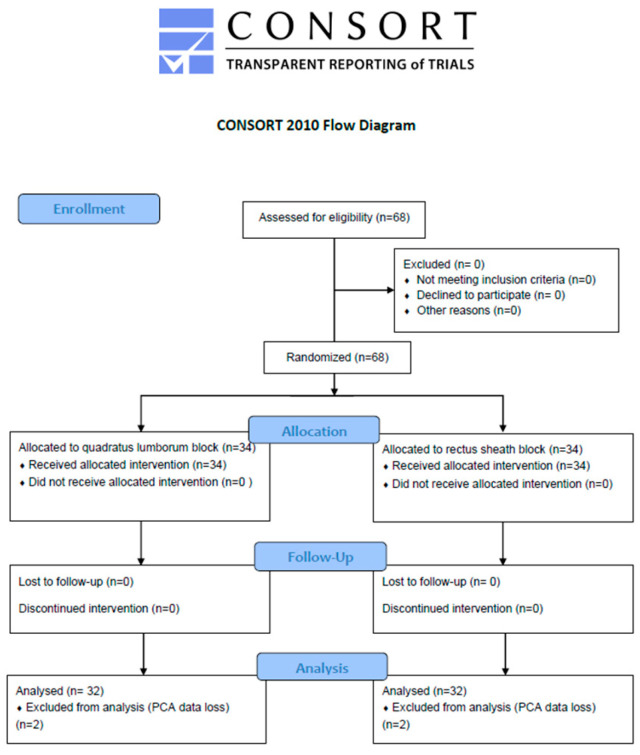
Consort Flow Diagram.

A total of 68 patients were enrolled in this study. Four were excluded due to missing PCA data—two in each group—resulting in 64 patients analyzed (QLB group, *n* = 32; RSB group, *n* = 32) (Figure 2). All patients included in the final analysis met the predefined criteria for block success, exhibiting at least partial sensory blockade (grade ≥ 1) in one or more dermatomes between T4 and L3. No block failures or block-related complications were observed. No patients were lost to follow-up, and no protocol deviations occurred during this study.

Baseline characteristics, including age, height, weight, BMI, surgical procedure, operation time, and anesthesia time, were comparable between the two groups (Table 1).

The primary outcome, cumulative opioid consumption during the first 24 h after surgery, was significantly lower in the RSB group (132.9 [61.3, 338.4] µg) compared to the QLB group (453.0 [253.1, 811.0] µg; *p* < 0.001). This difference was consistently observed at each postoperative time point. At 2, 4, 8, 12, 18, 32, and 48 h, the RSB group showed significantly lower cumulative opioid requirements compared to the QLB group (each comparison *p* < 0.001) (Table 2). Interval-based opioid consumption was also lower in the RSB group during most postoperative periods. Significant differences were noted from 0 to 24 h and again during the 32–48 h interval (each comparison *p* < 0.05), while no significant difference was found in the 24–32 h interval. Rescue analgesia with intravenous tramadol (25 mg) was administered to 10 patients (31.3%) in the QLB group and 4 patients (12.5%) in the RSB group during the 48-h postoperative period. This difference was not statistically significant (*p* = 0.061). These administrations were converted into fentanyl equivalents and included in the total opioid consumption analysis.

**Figure 3:**
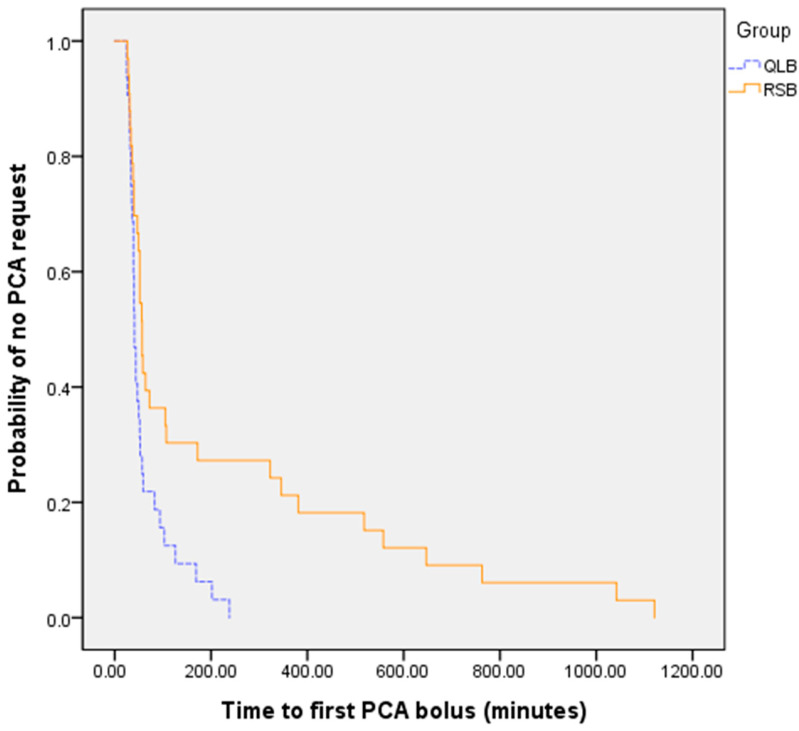
Kaplan–Meier survival curves showing the time to first patient-controlled analgesia bolus in the QLB and RSB groups. The RSB group exhibited a delayed first PCA request compared to the QLB group, indicating prolonged initial analgesic effect. A significant difference was observed between the two groups (log-rank *p* = 0.007). PCA: patient-controlled analgesia.

The time to first PCA bolus was significantly longer in the RSB group (56.5 [41.0, 340.3] minutes) than in the QLB group (40.5 [33.3, 68.8] minutes; *p* = 0.014), indicating a more prolonged initial analgesic effect. Kaplan–Meier survival analysis further confirmed a statistically significant delay in time to first PCA bolus in the RSB group (log-rank *p* = 0.007) (Figure 3).

Pain scores assessed by the VAS remained low and comparable between the groups at all postoperative time points. There were no statistically significant differences at any time from 2 to 48 h postoperatively (Table 3).

The incidence of PONV during the first 48 h was 15.6% (*n* = 5) in the QLB group and 21.2% (*n* = 7) in the RSB group (*p* = 0.562). Apfel scores were comparable, suggesting similar baseline risk for PONV between the groups.

## 4. Discussion

In this randomized controlled trial comparing QLB and RSB for postoperative analgesia in single-port laparoscopic adnexal surgery, RSB demonstrated superior opioid-sparing effects. The cumulative opioid consumption during the first 24 h after surgery—the primary outcome—was significantly lower in the RSB group. Furthermore, RSB was associated with reduced opioid requirements during the early postoperative period (up to 18 h) and a significantly longer time to first PCA bolus. These findings suggest that RSB may provide more efficient early postoperative analgesia than QLB in this surgical setting. These results were contrary to our initial hypothesis, which anticipated that QLB would provide superior analgesia. This expectation was based on QLB’s broader anatomical coverage (T8–L1) and its reported ability to influence both somatic and visceral pain components through sympathetic pathway modulation. Given that QLB encompasses the T10–T11 dermatomes—the key sensory distribution involved in umbilical trocar placement—and may extend its effect to visceral structures, it was expected to outperform RSB, which provides more localized somatic blockade.

However, our findings indicate that in the context of single-port adnexal surgery, RSB provided more consistent and effective early postoperative analgesia. We explored several plausible explanations for the unexpectedly superior performance of RSB observed in this study.

First, the relatively limited visceral component in adnexal surgeries, such as ovarian cystectomy or adnexectomy, may explain the comparable or superior performance of RSB. Unlike hysterectomy, which often involves substantial manipulation of deep pelvic structures, adnexal surgery is generally associated with less visceral nociception [19]. As such, a block that effectively targets somatic pain in the anterior abdominal wall—such as RSB—may be sufficient for pain control. In our study, both QLB and RSB achieved consistent sensory blockade of the T10–T11 dermatomes, which correspond to the umbilical port site. However, RSB provided more immediate and concentrated somatic coverage, potentially resulting in faster and more reliable analgesia.

Second, the sensory blockade provided by QLB may have been less consistent and less intense than expected. Unlike certain peripheral nerve blocks that yield complete sensory loss within a defined region, QLB often produces only partial attenuation of pain sensation relative to unaffected areas rather than a complete block. This variability may stem from the complex, plexus innervation of the abdominal wall, where blocking a single nerve does not necessarily ensure complete dermatome coverage [20,21,22,23]. Moreover, as QLB is a fascial plane block, its efficacy largely depends on the diffusion pattern of the local anesthetic within the thoracolumbar fascial layers. Variability in tissue planes and individual anatomy can lead to uneven spread of the anesthetic, resulting in inconsistent blockade of target nerves [22,23,24,25,26].

Third, the superior analgesic efficacy of RSB observed in our study may be attributed to anatomical and physiological differences in local anesthetic spread. In the immediate postoperative period, patients remain in a supine position for an extended duration, limiting muscle movement and contraction. This reduction in dynamic tissue activity may hinder the spread of local anesthetic, particularly to the TPVS. In living subjects, muscle contractions and changes in tissue pressure are believed to facilitate the diffusion of local anesthetic; however, these mechanisms are likely diminished in the early postoperative period when patients are immobilized [27]. Furthermore, previous studies have suggested that prone positioning facilitates local anesthetic spread into the TPVS due to gravitational assistance, whereas in the supine position, gravitational counteraction may restrict diffusion toward the TPVS [28].

Although some early anatomical studies have reported TPVS spread following QLB, more recent findings suggest that such spread may be inconsistent. For instance, Flynn et al. [29] performed thoracic paravertebral blocks in 10 cadavers using methylene blue dye and observed TPVS spread in most cases, but no dye was found in the space anterior to the quadratus lumborum muscle or around the psoas major. Similarly, Kumar et al. [30] used contrast imaging in fresh cadavers and found no spread from the QLB space to the TPVS. Yang et al. [31] also reported that clear TPVS spread was not observed when comparing different QLB approaches. Collectively, these studies suggest that the spread of local anesthetic to the TPVS during QLB may be variable or limited, which could explain the inconsistent visceral analgesia provided by QLB in clinical practice.

While no previous studies have directly compared RSB and QLB in adnexal surgery, several indirect findings from gynecologic procedures support our results. In line with our findings, previous studies have reported limited analgesic efficacy of QLB in gynecologic surgery. In a randomized controlled trial by Chung et al. [32], QLB did not significantly reduce cumulative fentanyl consumption or pain scores following single-port laparoscopic hysterectomy. Similarly, Hansen et al. [33] reported no difference in opioid consumption between the QLB and control groups in patients undergoing total laparoscopic hysterectomy. In another study, Fujimoto et al. [34] found no significant improvement in the quality of recovery or postoperative pain following QLB after gynecologic laparoscopic surgery. These findings suggest that the analgesic benefits of QLB may not be consistent across all surgical settings, particularly when used as a single-shot block in minimally invasive gynecologic procedures.

On the other hand, our findings are supported by previous reports highlighting the efficacy of RSB in gynecologic surgeries. In a randomized trial by Cho et al. [35], patients who received bilateral RSB during laparoscopic gynecologic surgery showed significantly lower fentanyl consumption in the PACU, prolonged time to first rescue analgesic, and a reduced number of rescue doses administered during the 48-h postoperative period. Similarly, Choi et al. [36] reported that in single-port total laparoscopic hysterectomy, fentanyl use at 8 h postoperatively was significantly lower in the RSB group (148 ± 61 µg) compared to the control group (222 ± 107 µg). Additionally, RSB has been shown to improve recovery quality after open midline gynecologic surgery [37].

Taken together, these findings suggest that for single-port laparoscopic adnexal surgery—where visceral pain is limited and somatic pain predominates—RSB may offer more consistent and opioid-sparing analgesia than QLB. Given its simplicity, reproducibility, and reliable dermatomal coverage, RSB may serve as a practical and efficient regional analgesic technique to optimize postoperative pain control in this specific surgical context.

However, these results should be interpreted with caution when applied to procedures involving greater visceral nociception, broader surgical fields, or different port configurations, such as multiport laparoscopy or open abdominal surgery. Further studies are warranted to confirm these findings in more diverse surgical populations.

Moreover, as this was a single-center study, institutional variability in surgical and analgesic practices may limit the external generalizability of our results. Interestingly, despite a significantly higher opioid consumption in the QLB group, the incidence of PONV did not differ significantly between the groups. One possible explanation for this finding is the uniform administration of prophylactic antiemetic combination therapy—dexamethasone and a 5-HT_3_ receptor antagonist—in both groups. This strategy reflects recommendations from the PROSPECT guideline for laparoscopic gynecologic surgery. Although specific protocols for single-port adnexal surgery are lacking, the consistent application of this preventive regimen may have attenuated the expected opioid-related differences in PONV outcomes. Nevertheless, other contributing factors cannot be excluded.

An unexpected finding in our study was that the RSB group continued to show significantly lower opioid consumption during the 32–48 h interval, despite the fact that the analgesic effects of a single-shot block with 0.375% ropivacaine are typically expected to wear off by 24 h. This prolonged difference may reflect various secondary factors. One possibility is that the higher opioid use in the QLB group during the early postoperative period could have contributed to tolerance or altered pain perception on the second day. Alternatively, the more consistent and effective early analgesia provided by RSB may have helped prevent the development of central sensitization or hyperalgesia, resulting in less pain and opioid need beyond the block’s expected duration. Although all surgical and baseline characteristics were comparable between groups due to randomization, and no systematic difference in surgical extent is expected, other unmeasured behavioral or physiological factors—such as earlier mobilization or reduced inflammation at the wound site—might also have played a role. Lastly, this finding may simply reflect a statistical anomaly or late-onset rebound pain in some QLB patients. Further investigation is warranted to better understand the mechanisms underlying this sustained opioid-sparing effect.

To the best of our knowledge, this is the first randomized controlled trial to directly compare the analgesic efficacy of QLB and RSB specifically in the setting of single-port laparoscopic adnexal surgery. This direct comparison provides valuable data to guide block selection for this surgical context, where no prior head-to-head studies exist. Additionally, although several previous studies have investigated the effects of QLB or RSB in gynecologic surgeries, they have typically included heterogeneous surgical populations encompassing procedures such as hysterectomy, myomectomy, and adnexal surgery [35,37,38]. These procedures vary substantially in their degree of visceral pain, and combining them in a single analysis may obscure the true analgesic effect of regional techniques. By focusing exclusively on single-port adnexal surgery—a relatively homogeneous group characterized by predominantly somatic pain—our study provides procedure-specific evidence that may help refine postoperative analgesia strategies for this increasingly utilized minimally invasive approach.

This study has several limitations. First, we did not separately assess somatic and visceral components of postoperative pain. Since adnexal surgery involves both somatic pain from trocar site incisions and visceral pain originating from the ovary and associated structures, distinguishing between these types of pain could have provided a more accurate understanding of the respective contributions of QLB and RSB to postoperative analgesia. As a result, we cannot determine whether QLB provided any visceral analgesia that went unrecognized due to the dominant contribution of somatic (incisional) pain in this surgical setting. While single-port adnexal surgery likely generates minimal visceral pain compared to more invasive procedures such as hysterectomy, some degree of visceral nociception—such as peritoneal stretch or ovarian traction—may still be present. However, our study was not designed to isolate or assess these pain components individually. Therefore, our findings primarily reflect overall analgesia and should not be interpreted as evidence that QLB is ineffective for visceral pain. Caution should be exercised in generalizing these results to procedures associated with more prominent visceral pain components.

Second, individual variability in pain perception and PCA usage patterns may have influenced opioid consumption, potentially affecting the objectivity of our comparisons.

In addition, our selection of the QLB approach may be considered a methodological limitation. The posterior QLB used in our study may have limited spread to the TPVS, potentially resulting in inadequate blockade of the ventral rami of thoracic spinal nerves T9–T11. This could have led to suboptimal analgesia in the midline region, including the umbilicus, where the surgical incision was centered. In contrast, transmuscular QLB is more likely to achieve TPVS spread and effectively block these dermatomes. Recently, the supra-arcuate ligament block has been introduced as a promising alternative that may facilitate more consistent anterior spread of local anesthetic into the TPVS by targeting the fascial plane above the medial arcuate ligament [39,40]. This approach theoretically enhances blockade of thoracic spinal nerves and may offer advantages in terms of anatomical coverage and safety. Therefore, caution is warranted when interpreting these findings, as the limited spread of posterior QLB could be a contributing factor to its reduced effectiveness in this setting.

However, these anatomical assumptions are not yet supported by definitive clinical evidence. To date, no QLB approach has demonstrated consistent superiority over others in randomized clinical trials or meta-analyses. We selected the posterior approach based on its practical and clinical advantages. Notably, it allows the block to be performed after surgery and before emergence from anesthesia, maximizing early postoperative analgesia without the need for lateral positioning. This minimizes operating room turnover time and avoids risks associated with patient repositioning under general anesthesia, such as airway stimulation or disconnection of lines. Additionally, transmuscular QLB requires needle advancement through multiple anatomical layers to reach the interfascial plane between the quadratus lumborum and psoas major muscles. This region lies in close proximity to the lumbar plexus, and local anesthetic spread—either inadvertently via needle trajectory or passively through porous fascia—may reach motor branches such as the femoral, obturator, or lateral femoral cutaneous nerves [41,42,43,44]. This carries the potential for lower limb motor weakness, which is incompatible with early ambulation and discharge goals in fast-track gynecologic recovery protocols.

Another limitation of this study is the absence of patient blinding. Although outcome assessors were blinded to group allocation, patients and anesthesiologists were not. Complete blinding was inherently difficult due to the anatomical differences between block sites. Both interventions were performed bilaterally under general anesthesia, which likely minimized patients’ awareness of the specific block received. However, since we did not employ sham injections or apply identical dressings to both potential block sites, full patient blinding could not be ensured. This limitation may have introduced some degree of bias in subjective outcomes such as pain scores and PCA usage.

In addition, this study did not include a no-block or placebo control group, as both QLB and RSB were actively administered. While this allowed a direct comparison between the two techniques, it limits our ability to assess the absolute analgesic benefit of each block relative to standard care.

Our study population consisted of relatively healthy, opioid-naïve women (ASA physical status I–III) undergoing benign adnexal surgeries. Patients with higher BMI, chronic opioid use, or malignant disease were excluded. These inclusion criteria enhance internal validity but may limit the generalizability of the findings to broader or more medically complex populations.

All procedures were performed at a single center by a consistent surgical and anesthesiology team. Although this standardization supports procedural consistency, it may reflect an optimal clinical scenario and reduce external applicability due to the single-center design and small sample size. Furthermore, the follow-up period was limited to 48 h postoperatively. Long-term outcomes such as functional recovery, the length of hospital stay, or the development of chronic pain were beyond the scope of this study.

Lastly, the findings are specific to the block techniques and drug regimens used—namely, bilateral QLB and bilateral RSB with 20 mL of 0.375% ropivacaine per side. Variations in technique, dosage, or the use of adjuvants may produce different results and warrant further investigation [24].

Future studies should aim to differentiate somatic and visceral components of postoperative pain to better clarify the specific analgesic contributions of QLB and RSB. Additionally, imaging-based verification of local anesthetic spread could help correlate block effectiveness with anatomical coverage. Expanding the research to other types of minimally invasive gynecologic procedures, higher-risk patient populations, or incorporating long-term recovery outcomes would further support the development of procedure-specific analgesic protocols. No block-related complications were observed in either group. Both QLB and RSB are generally considered safe under ultrasound guidance, although rare adverse events have been reported. For RSB, these include extrarectus sheath injection, vascular injury, bowel injury, and local anesthetic systemic toxicity (LAST) [45]. QLB has been linked to quadriceps weakness, retroperitoneal hematoma, hypotension, hoarseness, and LAST [39,46]. While uncommon, these risks highlight the importance of proper technique and patient selection. Larger studies are needed to further confirm their safety profiles in broader populations.

## 5. Conclusions

RSB was associated with significantly lower 24-h opioid consumption and a longer time to first PCA bolus compared to QLB in single-port laparoscopic adnexal surgery. Despite comparable pain scores between groups, these findings suggest that RSB may offer more efficient analgesia—achieving similar pain relief with reduced opioid requirements—particularly in procedures where visceral pain is limited. These results support considering RSB as a preferred regional technique in SPA-based adnexal surgeries to minimize opioid use.

## Figures and Tables

**Table 1:** Clinical characteristics.

	QLB Group	RSB Group	*p*-Value
Age (yr)	38.7 ± 11.9	38.3 ± 15.3	0.614
Height (cm)	161.0± 6.0	159.3 ± 5.6	0.132
Weight (kg)	64.3 ± 12.9	61.7 ± 9.8	0.067
BMI (kg/m^2^)	25.1 ± 4.3	24.3 ± 3.8	0.258
Operation time (minutes)	78.9 ± 39.9	72.3 ± 32.0	0.554
Anesthesia time (minutes)	115.6 ± 43.7	109.7 ± 32.8	0.364
ASA (I/II/III)	21/10/1	18/14/0	0.439
Type of surgery			
Ovary cystectomy (Unilateral/bilateral)	4/7	3/8	
Adnexectomy (Unilateral/bilateral)	17/4	18/3	

Baseline demographic and clinical characteristics of patients who underwent single-port laparoscopic adnexal surgery. Data are presented as mean ± standard deviation. QLB: quadratus lumborum block, RSB: rectus sheath block.

**Table 2:** Opioid Consumption.

	QLB Group (*n* = 32)	RSB Group (*n* = 32)	*p*-Value
Interval Opioid consumption (µg)			
0–2 h	98.8 [38.1, 147.5]	33.5 [25.0, 67.5]	0.001 *
2–4 h	62.4 [30.6, 99.4]	0.0 [0.0, 27.5]	<0.001 *
4–8 h	62.5 [32.7, 143.4]	0.0 [0.0, 33.4]	<0.001 *
8–12 h	32.8 [0.0, 99.4]	25.8 [0.0, 50.5]	0.024 *
12–18 h	68.5 [6.3, 207.2]	30.0[0.0, 74.5]	0.036 *
18–24 h	89.6 [30.6, 149.9]	25.2 [0.0, 75.7]	0.004 *
24–32 h	65.6 [0.0, 120.0]	0.0 [0.0, 74.1]	0.055
32–48 h	35.9 [0.0, 99.4]	0.0 [0.0, 27.2]	0.003 *
Cumulative opioid consumption (µg)			
2 h	98.8 [38.1, 147.5]	33.5 [25.0, 67.5]	0.001 *
4 h	142.2 [76.3, 240.4]	53.9 [25.0, 96.3]	<0.001 *
8 h	221.1 [117.9, 383.5]	57.5 [25.6, 113.8]	<0.001 *
12 h	281.3 [146.1, 473.2]	95.0 [28.1, 140.0]	<0.001 *
18 h	378.6 [180.0, 741.3]	111.1 [43.8, 258.1]	<0.001 *
24 h	453.0 [253.1, 811.0]	132.9 [61.3, 338.4]	<0.001 *
32 h	546.1 [269.4, 899.8]	164.5 [69.4, 357.5]	<0.001 *
48 h	690.1 [278.8, 1031.6]	164.5 [69.4, 358.8]	<0.001 *

Opioid consumption (µg) over time following single-port adnexal surgery in patients who received either QLB or RSB. Data are presented as median [Q1, Q3]. A *p*-value < 0.05 indicates a statistically significant difference between groups. QLB: quadratus lumborum block, RSB: rectus sheath block, *: *p*-value < 0.05.

**Table 3:** Pain score.

	QLB Group (*n* = 32)	RSB Group (*n* = 32)	*p*-Value
Pain score (VAS)			
2 h	2.0 [2.0, 3.0]	2.0 [2.0, 2.0]	0.456
4 h	2.0 [2.0, 2.0]	2.0 [2.0, 3.0]	0.087
8 h	2.0 [2.0, 2.8]	2.0 [2.0, 3.0]	0.313
12 h	2.0 [2.0, 2.0]	2.0 [2.0, 2.0]	0.712
18 h	2.0 [2.0, 2.0]	2.0 [2.0, 2.0]	0.958
24 h	2.0 [2.0, 2.0]	2.0 [2.0, 2.0]	0.981
32 h	2.0 [2.0, 2.0]	2.0 [2.0, 5.3]	0.125
48 h	2.0 [2.0, 2.0]	2.0 [2.0, 2.0]	0.326
Time to first bolus (minutes)	40.5 [33.3, 68.8]	56.5 [41.0, 340.3]	0.014 *

Pain scores and time to first bolus are presented as median [Q1, Q3] and were compared using the Mann–Whitney U test due to non-normal distribution. A *p*-value < 0.05 indicates statistical significance. QLB: quadratus lumborum block, RSB: rectus sheath block, VAS: visual analogue scale. *: *p*-value < 0.05.

## Data Availability

The datasets used and/or analyzed during the current study are available from the corresponding author on reasonable request.
